# Effectiveness of nutrition support team-led care on perioperative outcomes in malnourished older adults with gastric cancer

**DOI:** 10.3389/fnut.2025.1707892

**Published:** 2025-12-10

**Authors:** Lulu Qiu, Zhe Lin, Le Yang, Miao Li, Lichun Cheng

**Affiliations:** 1 Department of Pharmacy, The Second Affiliated Hospital of Dalian Medical University, Dalian, China; 2 Office of the Ethics Committee, The Second Affiliated Hospital of Dalian Medical University, Dalian, China

**Keywords:** nutrition support team, malnutrition, gastric cancer, older adults, recovery

## Abstract

**Background:**

Malnutrition is a prevalent complication in older adults with gastric cancer and significantly impacts postoperative outcomes following curative gastrectomy. This study aimed to investigate the clinical value of nutrition support team (NST) in the perioperative management of gastric cancer older adults with concomitant malnutrition.

**Methods:**

This retrospective cohort study included patients aged ≥65 years who underwent curative gastrectomy and met the Global Leadership Initiative on Malnutrition (GLIM) criteria for malnutrition between 2021 and 2024. Outcomes were compared between the NST group and conventional nutritional management group to analyze differences in nutritional support efficacy and clinical outcomes.

**Results:**

NST group showed lower mortality at day 1 and 30 (0.0 vs. 0.4%, 0.8 vs. 4.6%, *P* < 0.05) and higher compliance for energy (71.4 vs. 10.0%, *P* < 0.001) and protein intake (56.0 vs. 10.8%, *P* < 0.001) compared to traditional nutrition (TN) group. Prognostic nutritional index (PNI) [46.60 (43.45, 50.05), *P* < 0.0001; 47.30 (43.45, 50.05), *P* < 0.0001] and prealbumin [136.8 (123.2, 172.0), *P* < 0.0001; 157.0 (128.2, 183.4), *P* < 0.0001] were significantly higher in NST group at day 7 and discharge. NST reduced the incidence of anastomotic leakage (1.7 vs. 5.0%, *P* < 0.05) and infection rates (4.5 vs. 10.4%, *P* < 0.05), weight loss at day 7 and before discharge [2.12 ± 0.10% (95% CI: 1.95, 2.28) vs. 6.63 ± 0.20% (95% CI: 6.23, 7.03), *P* < 0.001; 1.92 ± 0.07% (1.78, 2.07) vs. 6.53 ± 0.20% (6.13, 6.93), *P* < 0.001]. NST group had a shorter length of stay [15.00 (14.00, 17.00), *P* < 0.05], postoperative stay [12.00 (9.00, 14.00), *P* < 0.05], and lower readmission rates (10.8 vs. 17.8%, *P* < 0.05). NST significantly reduced the time to drain removal after surgery [9.00 (8.00, 11.00), *P* < 0.001], time to first flatus [3.00 (3.00, 3.00), *P* < 0.001] and bowel movement [4.00 (4.00, 4.00), *P* < 0.001] were shorter in NST group.

**Conclusion:**

Our results demonstrated that NST intervention was associated with superior postoperative survival outcomes in malnourished older adults with gastric cancer. These findings supported that NST may serve as a valuable component of routine perioperative care for this vulnerable population.

## 1 Introduction

Gastric cancer represents one of the most prevalent malignancies globally, with GLOBOCAN 2024 data ranking it fifth in both incidence and mortality (1). The disease demonstrates marked age-dependent distribution, with epidemiological projections indicating that patients aged ≥65 years will comprise 68.6% of all gastric cancer cases by 2040, representing a substantial increase from 58.9% in 2020 (2). Tumor-associated catabolism, reduced oral intake, and gastrointestinal dysmotility significantly increase malnutrition risk in gastric cancer patients compared to other solid malignancies, particularly among the older adults (3). Malnutrition manifests as involuntary weight loss, decreased serum prealbumin levels, and lymphocytopenia, among other features, which collectively compromise immune function and reduce tolerance to surgical intervention (4).

Despite significant technical advances in radical gastrectomy (5, 6), this procedure remains highly invasive and induces a profound hypercatabolic state accompanied by immunosuppression, thereby predisposing patients to malnutrition of varying severity. Based on the Global Leadership Initiative on Malnutrition (GLIM) criteria, the perioperative prevalence of malnutrition among older adults with gastric cancer approaches 70% (7). The pathophysiology of perioperative malnutrition follows a distinct temporal pattern: pre-existing nutritional deficits are exacerbated by intraoperative metabolic derangements and further compounded by postoperative hypercatabolism, a cascade that is particularly severe in geriatric populations (8). Substantial evidence demonstrates that malnutrition is associated with significantly increased rates of postoperative infectious complications, prolonged hospital length of stay, and reduced long-term survival in this vulnerable cohort (9–11). Therefore, implementing comprehensive perioperative nutritional interventions to optimize recovery outcomes in older adults with gastric cancer represents a critical and unmet clinical need.

In response to this challenge, Nutrition Support Team (NST), multidisciplinary collaborative units based on evidence-based medicine, had emerged as critical components of modern healthcare systems for optimizing patient nutritional status (12, 13). By integrating specialized expertise from physicians, dietitians, pharmacists, and nurses, NSTs deliver individualized, precision-based nutritional interventions for malnourished patients. Strong evidence demonstrates that NST intervention significantly improves nutritional outcomes in hospitalized patients and reduces nutrition-related complications (14, 15). Following NST implementation, Kennedy et al. observed a substantial reduction in catheter-associated infection rates from 71 to 29%, along with a decrease in hospital length of stay by 2–4 days (16). A systematic review incorporating an economic model of 27 randomized controlled trials demonstrated that nutritional support yields average cost savings of $2,818 per patient (17).

However, evidence regarding perioperative NST implementation in malnourished older adults with gastric cancer remains limited, with both mechanistic insights and clinical benefits requiring further investigation. This study assessed the impact of NST-guided intervention on perioperative nutritional status, complications, and clinical outcomes in this population. By establishing optimal perioperative nutrition support strategies and promoting multidisciplinary collaboration in oncologic nutrition, this investigation aimed to provide robust evidence for improving prognosis and quality of life in this vulnerable patient cohort.

## 2 Method

### 2.1 Study design and participants

This retrospective cohort study was approved by the Ethics Committee of the Second Hospital of Dalian Medical University (approval number: KY2025-697-01, approved on September 1, 2025). We screened consecutive patients who underwent primary radical gastrectomy for gastric cancer at the Department of Gastrointestinal Surgery, Second Affiliated Hospital of Dalian Medical University, between January 2021 and September 2024.

#### 2.1.1 Participant selection and nutritional assessment

Selection of 1,086 patients with gastric cancer followed a 2-step approach based on established nutritional assessment frameworks (18). First, nutritional risk screening was conducted using the Mini Nutritional Assessment-Short Form (MNA-SF), a validated tool comprising six items [food intake decline, weight loss, mobility, psychological stress/acute disease, neuropsychological problems, and body mass index (BMI)] with a total score ranging from 0 to 14 points (19). Patients with MNA-SF scores ≤ 11 were identified as being at nutritional risk and proceeded to comprehensive malnutrition diagnosis (20).

Second, patients identified with nutritional risk underwent comprehensive malnutrition diagnosis using the GLIM criteria (21). The GLIM framework requires the presence of at least one phenotypic criterion and one etiologic criterion for malnutrition diagnosis. The phenotypic criteria assessed were: (1) non-volitional weight loss (>5% within the past 6 months or >10% beyond 6 months); (2) low BMI (< 20 kg/m^2^ for patients < 70 years or < 22 kg/m^2^ for patients ≥70 years); and (3) reduced muscle mass. The etiologic criteria included: (1) reduced food intake or assimilation ( ≤ 50% of energy requirements for >1 week, or any reduction for >2 weeks, or chronic gastrointestinal conditions affecting nutrient absorption); and (2) inflammation/disease burden (presence of acute disease/injury or chronic disease-related inflammation).

Enteral nutrition (EN) via feeding tube was prioritized when gastrointestinal function was adequate (presence of bowel sounds, absence of abdominal distention, and tolerance to trial feeding). Transition from EN to supplemental parenteral nutrition (SPN) occurred when: (1) EN alone could not meet 50% of calculated energy requirements for 7 days, or (2) gastrointestinal intolerance developed (severe nausea, vomiting, diarrhea, or abdominal distention). Transition from SPN to total parenteral nutrition (TPN) was indicated when: (1) EN was contraindicated (intestinal obstruction, severe ileus, or anastomotic leakage), or (2) combined EN and PN could not achieve nutritional targets. Transition from parenteral nutrition (PN) to EN was attempted when gastrointestinal function recovered, with progressive advancement of enteral feeding as tolerated.

#### 2.1.2 Inclusion and exclusion criteria

Subsequently, 512 patients with nutritional risk and malnutrition were enrolled, after applying inclusion and exclusion criteria. Inclusion criteria were: (1) age ≥65 years; (2) MNA-SF score ≤ 11; (3) confirmed malnutrition diagnosis based on GLIM criteria. Exclusion criteria were: (1) BMI ≥28 kg/m^2^ (to exclude patients with obesity, where nutritional management strategies differ substantially); (2) concurrent malignancies; (3) incomplete medical records. Traditional nutrition (TN) group (*n* = 241) and NST (*n* = 241) were followed by the propensity score matching (PSM). PSM was employed to mitigate potential biases when comparing the TN and NST groups.

#### 2.1.3 Grouping and interventions

**Figure 1:**
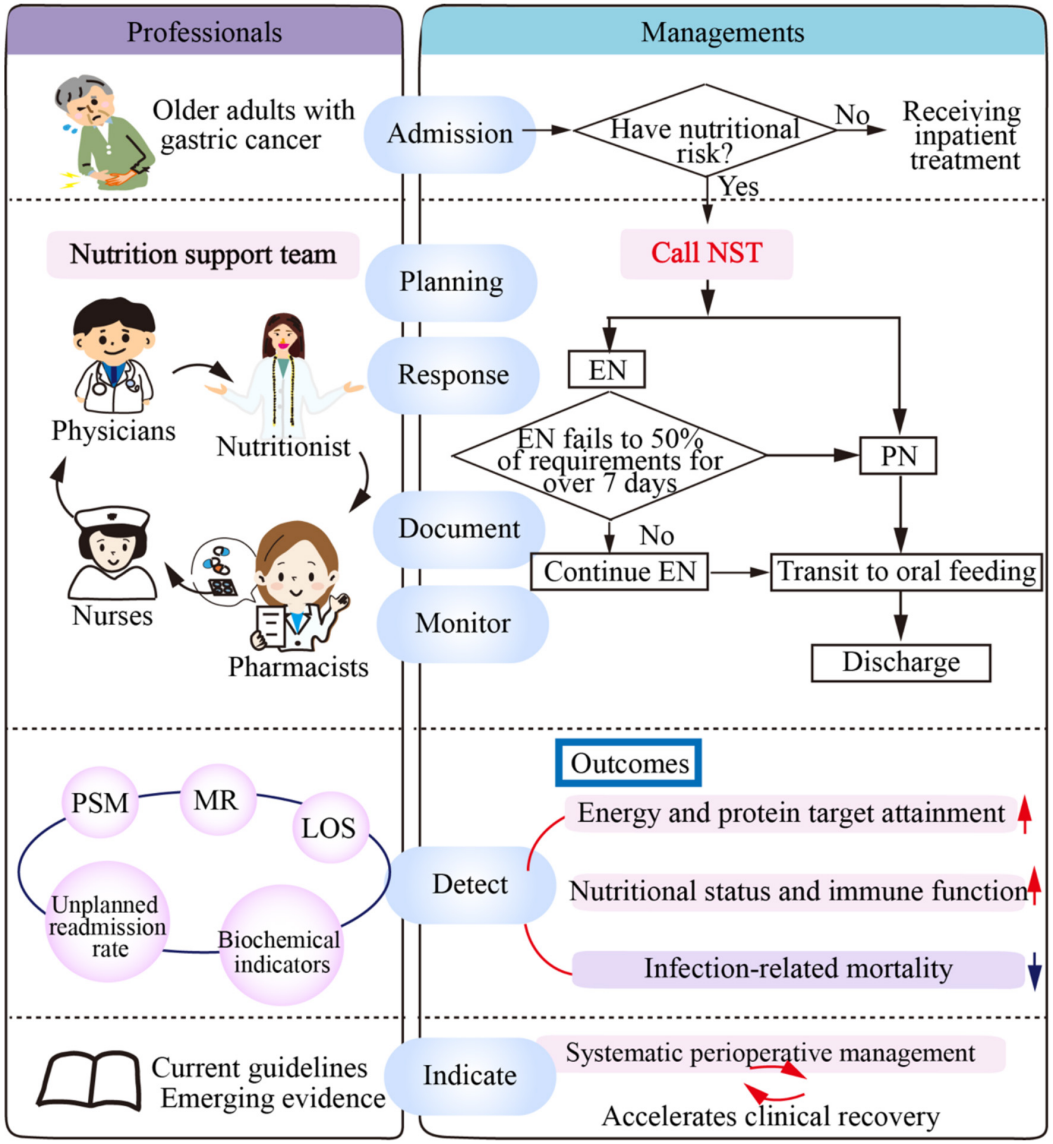
NST teamwork mode. NST teamwork mode for hospitalized older adults with gastric cancer: Nutritional risk is screened via Mini Nutritional Assessment-Short Form (MNA-SF)/Global Leadership Initiative on Malnutrition (GLIM) to confirm support needs. NST members collaborate on nutrition plans, monitor tolerance, electrolytes, and complications. Guided by evidence-based practices, the team analyzes key outcomes (30-day mortality, intake achievement, nutritional/immune improvement) to enable accelerated perioperative recovery. NST, nutrition support team; EN, enteral nutrition via feeding tube; PN, parenteral nutrition; PSM, propensity score matching; MR, mortality rate; LOS, length of stay.

The NST was established in January 2021, comprising a multidisciplinary team of healthcare professionals: a gastrointestinal surgeon, a registered dietitian, a clinical pharmacist, and a specialized nurse (Figure 1). Based on the nutritional intervention strategy employed, eligible patients were stratified into two groups. The TN group received conventional nutritional support, which consisted of routine dietary counseling and empirical nutritional supplementation prescribed by the attending physician in accordance with standard institutional protocols. In contrast, the NST group received comprehensive multidisciplinary nutritional care, including individualized nutritional assessment and intervention plans, along with regular follow-up throughout the perioperative period. Assignment to NST services was determined through a shared decision-making process involving the attending surgeon and the patient or their family members, with consideration given to the patient's willingness to participate in structured nutritional management and the availability of NST resources at the time of hospitalization.

### 2.2 Screening and baseline measurements

Preoperative baseline demographic and clinical characteristics of the patients were systematically documented, encompassing gender, age, body weight, height, BMI, surgical procedure (total gastrectomy or partial gastrectomy), surgical approach (laparoscopic or robot-assisted), duration of surgery, Charlson comorbidity Index (CCI), prognostic nutritional index (PNI), and MNA-SF score. Additionally, preoperative baseline biochemical parameters were measured, including levels of prealbumin, hemoglobin, and total bilirubin and creatinine, all of which were measured under fasting conditions. The preoperative body weight was defined as the body weight measured on the day of surgery. The body weight was then measured again at 1 month after surgery (postoperative weight) to calculate the percentage of postoperative weight loss (= [postoperative weight – preoperative weight]/preoperative weight × 100).

### 2.3 Process of NST activity

Clinical physicians initiated a 2-step GLIM protocol within 24 h of admission, consisting of nutritional risk screening and malnutrition diagnosis. For patients with confirmed malnutrition, a multidisciplinary team comprising physicians, dietitians, and clinical pharmacists developed individualized nutritional intervention plans. These evidence-based strategies were tailored to each patient's nutritional status, biochemical parameters, and gastrointestinal function, with specifications for caloric requirements and delivery routes. Registered nurses provided nutritional education, implemented standardized support protocols, and monitored and documented any adverse events.

For patients with confirmed malnutrition undergoing elective surgery, nutritional support was initiated preoperatively within 24–48 h of admission. For patients unable to resume oral intake within 48 h postoperatively, nutritional support therapy was promptly commenced. The NST conducted multidisciplinary rounds at least twice weekly to ensure appropriate implementation and safety of nutritional interventions.

Due to the impracticality of measuring individual energy expenditure, target energy intake was established at 25–30 kcal/kg/day and protein intake at 1.5 g/kg/day, following perioperative nutrition guidelines (22). The NST employed a progressive 5-tier nutritional support strategy: (1) oral intake (PO); (2) oral nutritional supplementation (ONS); (3) EN; (4) EN + SPN; and (5) TPN. When oral intake or ONS provided less than 50% of target energy requirements for 7 days, EN was initiated. When EN failed to meet more than 50% of patients' energy requirements within 7 days, SPN was commenced. For patients with contraindications to enteral nutrition, such as intestinal obstruction, severe paralytic ileus, or high-output enterocutaneous fistula, TPN was implemented immediately (23). PN formulations consisted of dextrose (50%−60% of non-protein calories), lipid emulsions (30%−40% of non-protein calories), and amino acids, with a non-protein calorie-to-nitrogen ratio of 100–150:1. All patients received vitamins and trace elements according to ASPEN guidelines (24), with daily electrolyte adjustments based on laboratory monitoring. Parenteral nutrition was administered via peripherally inserted central catheter lines.

Patient adherence to nutritional prescriptions was monitored by registered nurses through daily documentation of oral intake, enteral feeding volumes, and parenteral nutrition records. Adherence data were systematically reviewed and verified during multidisciplinary NST rounds. To ensure inter-rater reliability within the NST, standardized training was conducted prior to study initiation, unified assessment protocols were implemented, and consensus-based discussions were held during team meetings to minimize subjective variability in nutritional assessment and intervention decisions.

### 2.4 Outcomes

The primary outcome was the 30-day mortality rate following surgery. The diagnostic criterion for 30-day mortality was defined as any death from any cause occurring within 30 days of the day on which the patient underwent radical or palliative gastrectomy for gastric cancer. Secondary outcomes were assessed across three categories: (1) nutritional parameters: compliance rates for energy and protein intake targets were monitored throughout the perioperative period; postoperative nutritional status was evaluated using the PNI, along with serum levels of prealbumin, albumin, and hemoglobin measured on postoperative days 3 and 7, and at discharge; weight loss from baseline was documented for all patients; (2) surgical complications: anastomotic leakage rates were compared between groups; postoperative infections were defined according to the Centers for Disease Control and Prevention (CDC) criteria for healthcare-associated infections (25), with specific infectious complications assessed including pneumonia, intra-abdominal infection, urinary tract infection, and catheter-related bloodstream infection; and (3) recovery metrics: total hospital length of stay, postoperative length of stay, unplanned readmission rate, time to drain removal, time to first flatus, and time to first bowel movement were recorded.

### 2.5 Statistical analysis

Statistical analyses were conducted using SPSS version 27.0 for Windows (SPSS Inc., Chicago, IL, USA). Survival outcomes were estimated using the Kaplan–Meier method. PSM was employed to mitigate potential biases when comparing the TN and NST groups. A logistic regression model was fitted to estimate the propensity score, controlling for these covariates: gender, age, BMI, surgical type, operation, duration of surgery, prealbumin, hemoglobin, total bilirubin, creatinine, PNI, MNA-SF and Charlson comorbidity index (CCI). PSM was conducted using a 1:1 nearest-neighbor algorithm without replacement and a caliper width of 0.1. Successful balance between the groups was confirmed, with all absolute standardized mean differences (ASD) falling below the threshold of 0.2 post-matching. Continuous variables were assessed using the Kolmogorov-Smirnov test and compared using *t*-tests (normal distribution) or Mann–Whitney *U* test (abnormal distribution), with results expressed as mean ± standard error (SE) or median (P_25_, P_75_). Two-sided *P*-values < 0.05 were considered statistically significant. Categorical variables were compared using the χ^2^ test.

## 3 Results

### 3.1 Patient baseline characteristics

**Figure 2:**
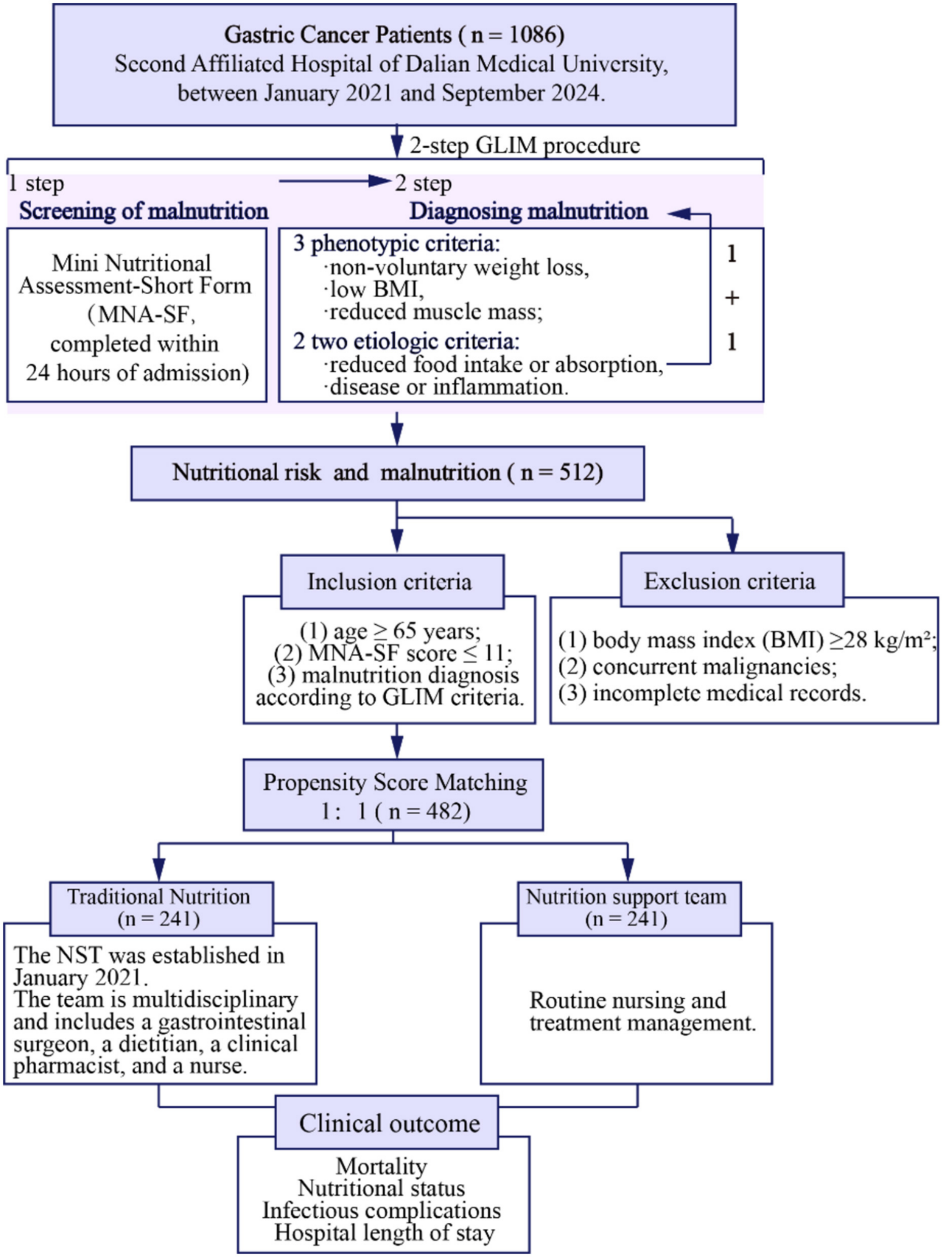
Flowchart. This retrospective cohort study enrolled gastric cancer patients ≥65 years undergoing radical gastrectomy (Jan 2021–Sep 2024) with Mini Nutritional Assessment-Short Form (MNA-SF) ≤ 11 and Global Leadership Initiative on Malnutrition (GLIM)-defined malnutrition. Exclusions: body mass index (BMI) ≥28 kg/m^2^, other malignancies, incomplete data. Two hundred and forty-one patients each in the nutrition support team (NST) group and traditional nutrition (TN) group.

A total of 513 patients consented to participate in this study between January 1, 2021, and December 30, 2024. After propensity score matching, 71 patients were excluded, leaving 482 patients (TN: 241, NST: 241) for analysis (Figure 2). No significant differences in baseline characteristics were observed between the two groups. The baseline characteristics of the included patients are presented in Table 1. Among the NST participants, the number of male participants is nearly twice that of female participants. Participants were on average about 70 years old. BMI was under 24 kg/m^2^. Regarding surgical procedures, threefold cases underwent partial gastrectomy rather than total gastrectomy, with twofold performed using robotic surgery. The mean duration of surgery was half-years. The values of nutritional indicators, renal function, bilirubin levels, and nutrition prognostic-related scores were comparable between the NST intervention group and the TN group.

**Table 1:** The comparison of general characteristics between the two group.

**Data**	**TN Value (95% CI)**	**NST Value (95% CI)**	**t/Chi-Square**	***P-*value**
**Gender (** ***n*** **, %)**				
Male	155, 64.3 (58.2, 70.4)	152, 63.1 (56.9, 68.2)	0.08	0.78
Female	86, 35.7 (29.6, 41.8)	89, 36.9 (31.8, 43.1)		
Age (year, means ± SE)	71.94 ± 0.34 (71.27, 72.61)	72.16 ± 0.35 (71.47, 72.85)	−0.45	0.65
BMI (kg/m^2^, means ± SE)	23.86 ± 0.18 (23.50, 24.21)	23.89 ± 0.18 (23.54, 24.25)	−0.14	0.89
**Surgical type (** ***n*** **, %)**				
Partial gastrectomy	189, 78.4 (73.19, 83.65)	189, 78.4 (73.19, 83.65)	0.00	1.00
Total gastrectomy	52, 21.6 (16.35, 26.81)	52, 21.6 (16.35, 26.81)		
**Operation (** ***n*** **, %)**				
Robot	76, 31.54 (25.63, 37.44)	75, 31.12 (25.23, 37.01)	0.10	0.92
Laparoscope	165, 68.46 (62.56, 74.37)	166, 68.87 (62.99, 74.77)		
Duration of surgery (minute, means ± SE)	189.71 ± 3.83 (182.16, 197.26)	188.34 ± 4.57 (179.33, 197.36)	0.23	0.82
Prealbumin (mg/L, means ± SE)	180.05 ± 2.86 (174.41, 185.69)	181.61 ± 2.89 (175.91, 187.31)	−0.38	0.70
Hemoglobin (g/L, means ± SE)	135.99 ± 1.62 (132.80, 139.18)	135.68 ± 1.62 (132.49, 138.88)	0.13	0.90
Total bilirubin (means ± SE, μmol/L)	13.31 ± 0.46 (12.41, 14.21)	13.19 ± 0.45 (12.30, 14.07)	0.19	0.85
Creatinine (means ± SE, μmol/L)	65.68 ± 1.14 (63.43, 67.92)	65.23 ± 1.10 (63.06, 67.40)	0.28	0.78
PNI (means ± SE)	43.47 ± 0.33 (42.82, 44.11)	43.52 ± 0.34 (42.85, 44.19)	−0.12	0.91
MNA-SF (means ± SE)	9.47 ± 0.05 (9.38, 9.57)	9.48 ± 0.05 (9.38, 9.58)	−0.12	0.90
CCI (means ± SE)	3.36 ± 0.10 (3.16, 3.56)	3.38 ± 0.10 (3.18, 3.58)	−0.18	0.86

TN, traditional nutrition; NST, nutrition support team; SE, standard error; BMI, body mass index; PNI, prognostic nutritional index; MNA-SF, mini nutritional assessment-short form; CCI, Charlson comorbidity index.

### 3.2 Prognosis

**Figure 3:**
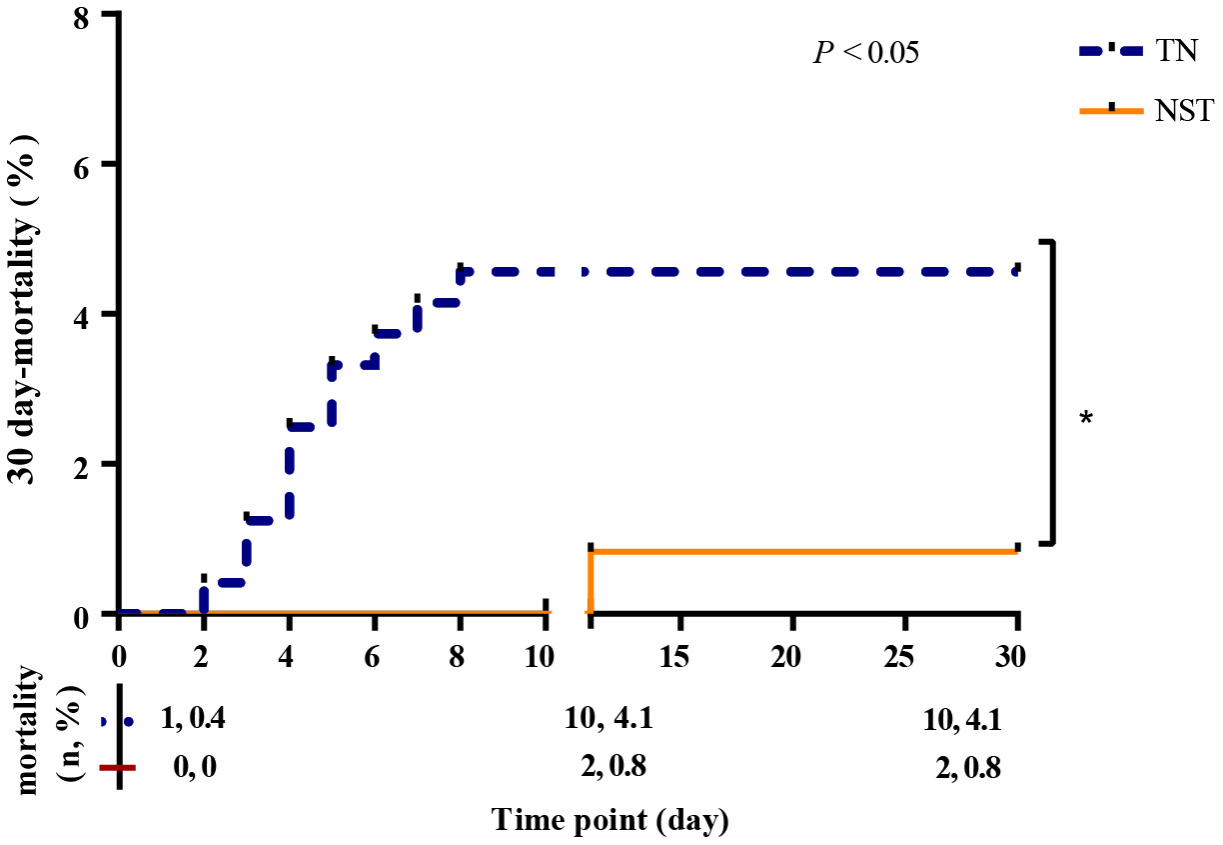
NST group declined the 30 day-mortality rates in malnourished older adults with gastric cancer. Thirty day-mortality of the TN group (*n* = 241; blue dash line) and NST group (*n* = 241; orange line). TN, traditional nutrition; NST, nutrition support team.

Figure 3 showed 30-day mortality rates curves. The mortality rates at day 1 and 30 were 0.4%−4.1% (95% CI: 1.9%−7.2%) in the TN group, compared to 0.0%−0.8% (95% CI: 0.0%, 2.0%) in the NST group, respectively (*P* < 0.05). Our data indicated that NST prominently declined the mortality rate of older adults with gastric carcinoma within 30 days. Among the 10 deaths in the TN group within 30 days postoperatively, the causes were septic shock (*n* = 6), respiratory failure (*n* = 1), heart failure (*n* = 1), pulmonary embolism (*n* = 1), and multiple organ dysfunction syndrome (MODS, *n* = 1). In the NST group, two deaths occurred, attributed to septic shock (*n* = 1) and MODS (*n* = 1).

### 3.3 Nutritional support outcomes

#### 3.3.1 Compliance rates for energy and protein intake

**Figure 4:**
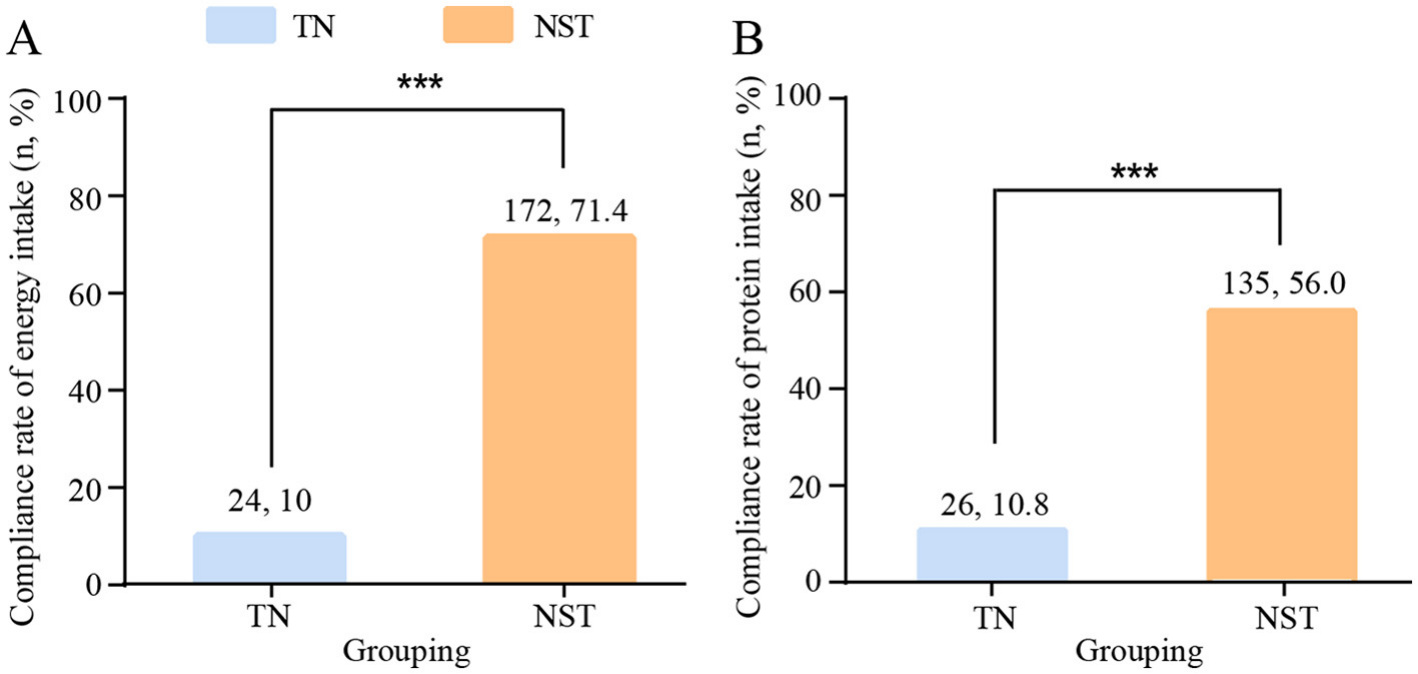
NST-led care boosted the compliance rates for **(A)** energy and **(B)** protein intake in malnourished older adults with gastric cancer. Compared to the TN group, ***P < 0.001. TN, traditional nutrition; NST, nutrition support team.

A total of 236 patients in the TN group and 221 patients in the intervention group received PN therapy. The compliance rates for energy and protein intake were compared between the two groups before discharge after gastrectomy, for supply a more standardized the nutritional support according to the ESPEN guidelines which calls for 25–30 kcal/kg/d energy supply and 1.2–1.5 g/kg/d protein intake. The NST group, compared with the TN group, showed a higher compliance rates for energy (71.4 vs. 10.0%, respectively, *P* < 0.001), and higher compliance rates for protein intake (56.0 vs. 10.8%, respectively, *P* < 0.001, Figure 4).

#### 3.3.2 Postoperative nutritional status

**Figure 5:**
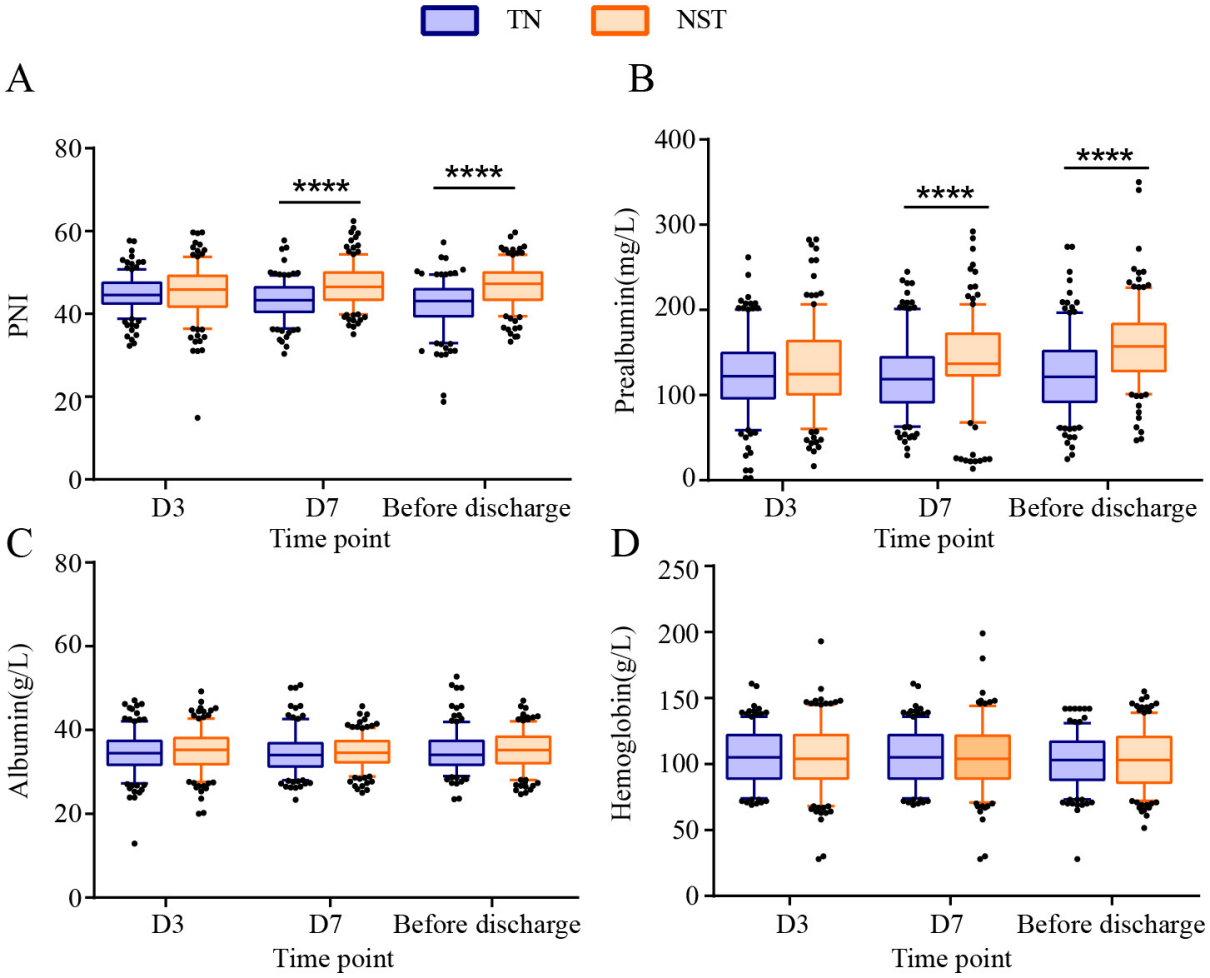
NST-led enhanced the postoperative nutritional status at day 3, 7 and before discharge in malnourished older adults with gastric cancer. Statistics analysis of **(A)** prognostic nutritional index (PNI), **(B)** prealbumin, **(C)** albumin and **(D)** hemoglobin. Compared to the TN group, ****P < 0.0001. PNI, prognostic nutritional index; TN, traditional nutrition; NST, nutrition support team.

The postoperative nutritional indicators PNI, prealbumin, albumin and hemoglobin were compared between the two groups at day 3, day 7 and before discharge, separately (Supplementary Table S1). The PNI were increased by NST group in comparison of the TN groups at day 7 and before discharge (*P* < 0.0001, Figure 5). These differences were clinically meaningful, as the mean improvements of 2.42 points at day 7 and 1.56 points before discharge elevated PNI above the critical threshold of 46, which was associated with reduced postoperative complication risk in gastric cancer patients (26). The prealbumin of NST group were observably elevated at day 7 and before discharge (*P* < 0.0001). The NST group reached prealbumin concentrations of approximately 150 mg/L before discharge, a threshold associated with adequate protein synthesis and reduced postoperative complications (27). The serum albumin level and hemoglobin were not significantly different between the TN and NST groups at day 3, day 7 nor before discharge.

#### 3.3.3 Postoperative complications

**Figure 6:**
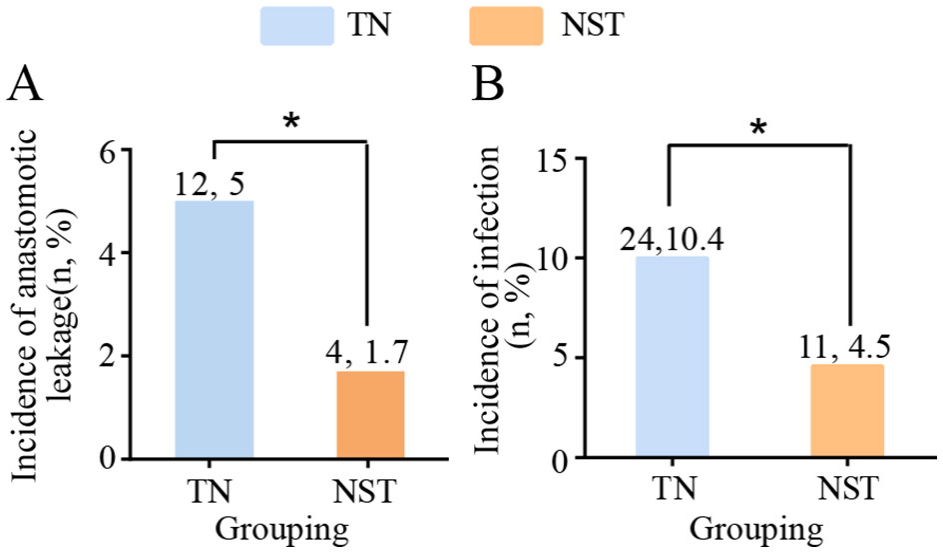
NST-led care impeded the postoperative complications before discharge in malnourished older adults with gastric cancer. Statistics of the number of patients with **(A)** anastomotic leakage and **(B)** incidence of infection. Compared to the TN group, *P < 0.05. TN, traditional nutrition; NST, nutrition support team.

Postoperative complications were shown in Figure 6. After NST intervention, the incidence of anastomotic leakage in older adults with gastric carcinoma was significantly decreased compared to the TN group (1.7 vs. 5.0%, *P* < 0.05). Similarly, infectious complications occurred less frequently in the NST group than in the TN group (4.5 vs. 10.4%, *P* < 0.05). A total of 23 infection episodes occurred in the TN group, comprising pneumonia (*n* = 13), catheter-related bloodstream infection (*n* = 1), abdominal infections (*n* = 7), and urinary tract infections (*n* = 2). In the NST group, 10 infection episodes were documented, including pneumonia (*n* = 5), abdominal infections (*n* = 4), and urinary tract infection (*n* = 1). In addition to anastomotic leakage and infectious complications, other major postoperative complications were also recorded. Intra-abdominal bleeding occurred in three patients (1.2%) in the TN group and two patients (0.8%) in the NST group. Chylothorax was observed in one patient (0.4%) in each group. Pulmonary embolism occurred in one patient (0.4%) in the TN group, with no cases documented in the NST group.

#### 3.3.4 Weight loss

**Figure 7:**
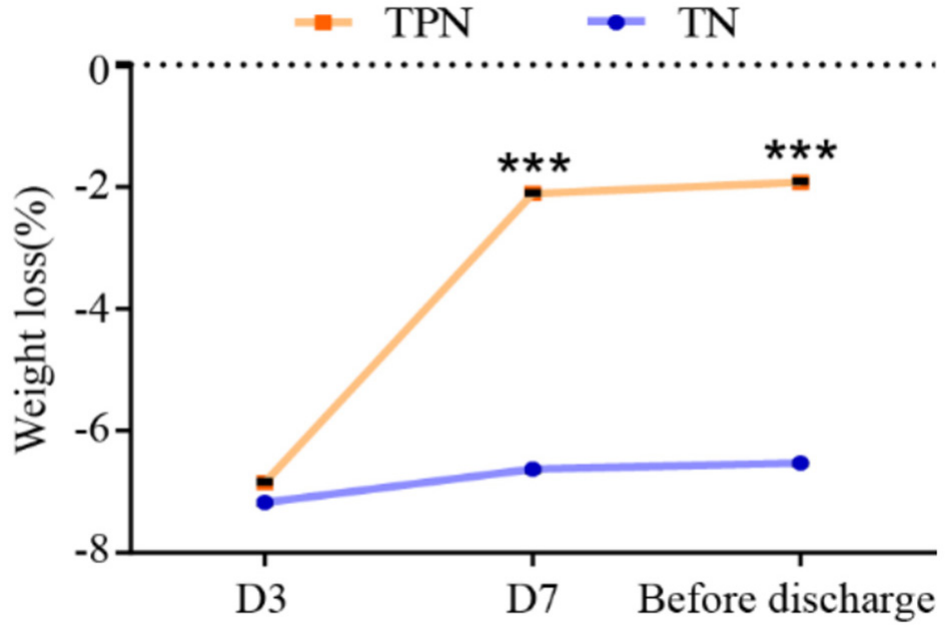
NST-led care rescued the perioperative weight loss in malnourished older adults with gastric cancer. Compared to the TN group, ***P < 0.001. TN, traditional nutrition; NST, nutrition support team.

A significant reduction of weight loss (D3: 2.11 ± 0.08 vs. 6.63 ± 0.20, *P* < 0.001; before discharge: 1.92 ± 0.07 vs. 6.53 ± 0.20, *P* < 0.001) in the NST-group was noted compared to the TN group at day 7 and before discharge (Figure 7).

#### 3.3.5 Recovery metrics

**Figure 8:**
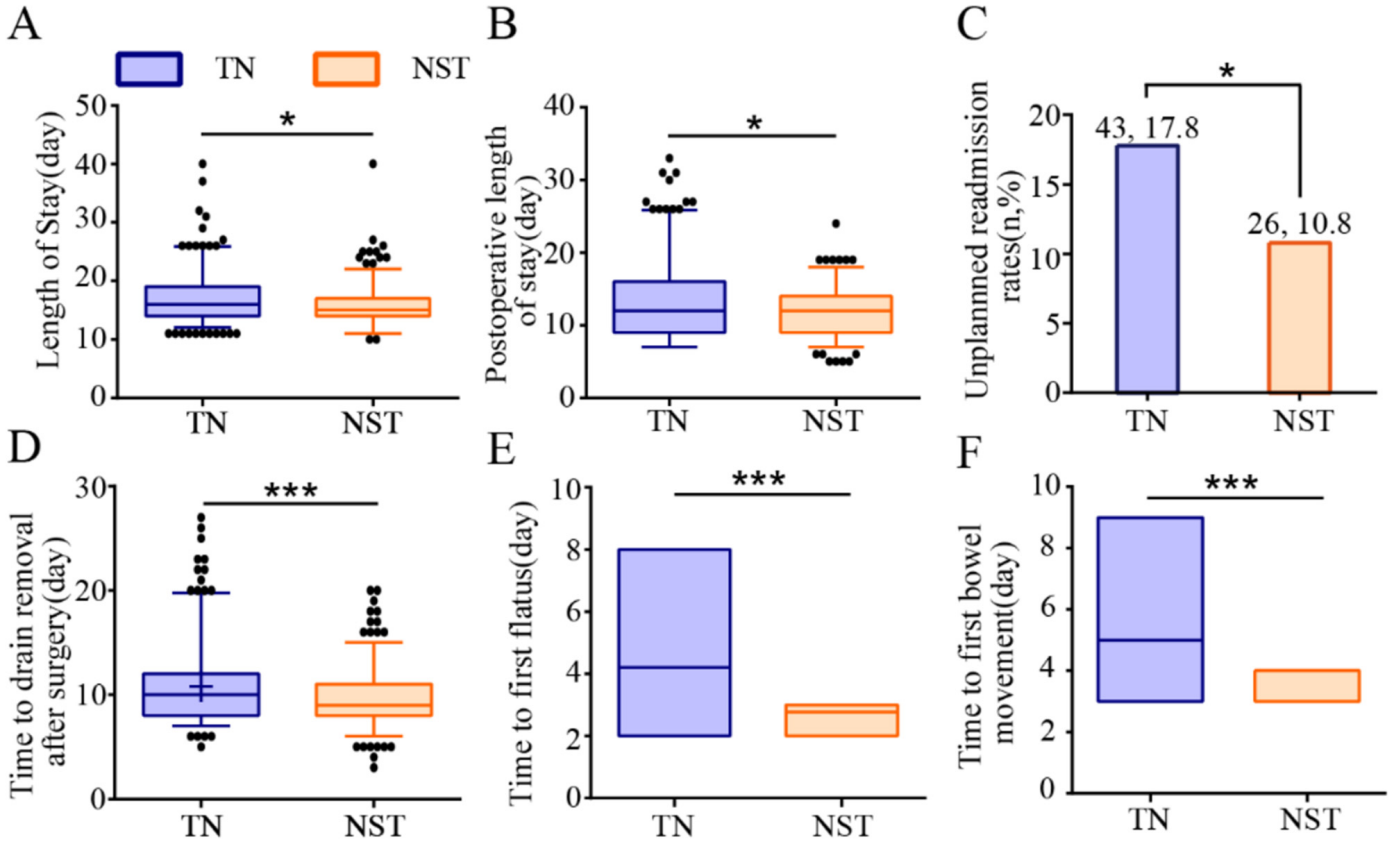
NST-led care improved recovery metrics in malnourished older adults with gastric cancer. Analysis of **(A)** length of stay, **(B)** postoperative length of stay, **(C)** unplanned readmission rates, **(D)** time to drain removal after surgery, **(E)** time to first flatus and **(F)** time to first bowel movement. Compared to the TN group, ***P < 0.001 or *P < 0.05. TN, traditional nutrition; NST, nutrition support team.

NST group significantly decreased the length of stay [15.00 (14.00, 17.00) vs. 16.00 (14.00, 19.00), *P* < 0.05] and postoperative length of stay [12.00 (9.00, 14.00) vs. 12.00 (9.00, 16.00), *P* < 0.05] and unplanned admission rate (10.8 vs. 17.8%, *P* < 0.05) compared to the TN group (Figure 8). Furthermore, NST significantly reduced the time to drain removal after surgery [9.00 (8.00, 11.00) vs. 10.00 (8.00, 12.00), *P* < 0.001], the first time to first flatus [3.00 (3.00, 3.00) vs. 4.00 (4.00, 4.00), *P* < 0.001] and first bowel movement [4.00 (4.00, 4.00) vs. 5.00 (5.00, 5.00), *P* < 0.001] compared with the TN group.

## 4 Discussion

This study evaluated the clinical value of NST in the perioperative management of malnourished older adults with gastric cancer. Our findings demonstrated that multidisciplinary NST involvement was associated with improved clinical outcomes through individualized nutrition regimens with continuous monitoring and adjustment. While the observational design of this study precludes definitive causal inference, the observed benefits followed a biologically plausible stepwise progression: adequate nutrient intake was associated with enhanced nutritional status, which correlated with reduced infection rates and shortened hospital stays. These improvements ultimately coincided with lower mortality, accelerated recovery, and improved quality of life. Our study was well-referenced with up-to-date literature and strong alignment with ESPEN and ASPEN guidelines.

These findings align with recent evidence demonstrating the beneficial impact of nutritional interventions in surgical populations. Schuetz et al. reported that individualized nutritional support to reach protein and caloric goals reduced the risk of adverse clinical outcomes in medical inpatients at nutritional risk (28), while Gomes et al. demonstrated that nutritional support interventions was associated with clinically significant improvements of important clinical outcomes in the medical inpatient population, in whom malnutrition is highly prevalent (29).

Causes of mortality in older adults with gastric cancer include postoperative malnutrition, infectious complications, age-related immunosuppression, and disease progression (11, 30, 31). The high prevalence of malnutrition in this population significantly increases the risk of postoperative complications and early mortality (32). Adequate caloric and protein supplementation represents a critical modifiable factor for improving prognosis (28, 33). In this study, the NST established evidence-based nutritional targets, which improving nutrition forms part of the ESPEN guidelines, recommending an energy supply of 25–30 kcal/kg/d and a protein intake of 1.2–1.5 g/kg/d (22). Through individualized nutritional prescriptions and a standardized titration protocol, the NST intervention group achieved significantly higher compliance rates for both energy and protein intake. Notably, conventional nutritional management alone proved insufficient to meet these targets (34). By comparison, the NST's systematic intervention ensures the effective delivery of nutritional therapy and underscores nutrition improvement as a global priority, a core element of United Nations Sustainable Development Goal 2 (35).

Malnutrition amelioration through enhanced nutrient intake correlates directly with improvements in biochemical parameters (36). Prealbumin, as a negative acute phase response protein with a short half-life, responds rapidly to nutritional interventions (37). Our results showed that the NST group exhibited a significant increase in prealbumin levels by postoperative day 7. Recent evidence has indicated that changes in prealbumin during the first week post-surgery can serve as an accurate predictor of patient mortality and are a reliable indicator for assessing the effectiveness of nutritional support (38, 39). Our findings suggest that NST may improve the nutritional status of patients, potentially contributing to a reduction in mortality rates. Additionally, the PNI first proposed by Onodera et al. (40), integrates serum albumin levels and peripheral blood lymphocyte counts and has been validated as an independent prognostic factor for gastrointestinal malignancies (41, 42). A significant increase in PNI was demonstrated in the NST group. PNI reflects both nutritional status and immune function, with its improvement being negatively correlated with the incidence of postoperative complications (43–45). Our findings indicated that NST facilitated the effective recovery of nutritional status and immune function.

Infectious complications constitute a major source of perioperative morbidity and mortality in older adults with gastric cancer (46), with their incidence being closely associated with patients' nutritional status (47, 48). The findings of this study demonstrated that the incidence of infectious complications was significantly lower in the NST intervention group compared to the TN group, consistent with results reported in previous studies (49, 50). Notably, NST intervention significantly decreased all-cause mortality in the subset of patients with infectious complications. Malnourished patients are more susceptible to progression from infection to sepsis (51), while NST enhanced patients' resistance to infectious insults by improving nutritional status. Furthermore, optimal nutritional support preserved the integrity of the intestinal barrier, reduced bacterial translocation, and mitigated the risk of sepsis-related mortality (52).

The superior infection control in the NST group reflects multiple interconnected immunological mechanisms. Adequate protein supports immunoglobulin synthesis and lymphocyte proliferation (49), with specific amino acids (glutamine, arginine) enhancing immune cell function and maintaining intestinal barrier integrity (53, 54). Sufficient energy intake maintains neutrophil and macrophage phagocytic capacity (52), while omega-3 fatty acids modulate inflammatory responses toward anti-inflammatory pathways (55). Critical micronutrients serve as immune cofactors: zinc enables T-cell maturation (56), selenium provides antioxidant protection (57), vitamin D induces antimicrobial peptides (58), and vitamin C supports phagocyte function (59).

NST protocols emphasizing early enteral nutrition preserve gut-associated lymphoid tissue (60), maintain microbiota diversity, and prevent bacterial translocation (61, 62), Comprehensive nutritional support restores antioxidant defenses, particularly important in older adults with immunosenescence (63). The multidisciplinary NST approach provides distinct advantages: individualized assessment prevents both underfeeding and overfeeding complications (64), early intervention optimizes the post-operative immune window (47). These synergistic mechanisms, including macronutrient-driven energy restoration, micronutrient-enhanced immune signaling, and gut health maintenance, ultimately reduce infection complications and improve clinical outcomes (65).

Maintaining body weight independently and significantly predicts the prognosis of surgical patients (66). Numerous previous studies have consistently demonstrated a strong correlation between perioperative unintentional weight loss and increased rates of postoperative complications, prolonged hospital stays, and higher mortality (67–69). In the present study, our results indicated that unintentional weight loss in patients receiving NST interventions was significantly lower than that in the TN group, which has considerable clinical significance. It has been reported that nutritional support establishes a necessary metabolic foundation for the body to cope with surgical trauma stress and promotes postoperative recovery by preserving lean body mass and maintaining the reserve function of visceral protein pools (70). This finding further underscores the critical value of individualized perioperative nutritional support strategies in improving the prognosis of surgical patients.

The accelerated recovery of gastrointestinal function and the reduced length of hospital stay are comprehensive indicators reflecting all of the aforementioned improvements (71). In this study, we observed that the time to first postoperative flatus and defecation was significantly shortened in the intervention group. This can be attributed not only to the direct facilitative effect of adequate energy and protein supplementation on intestinal mucosal repair (47), but also to the attenuation of systemic inflammatory responses and reduction in infectious complications as a result of improved nutritional status (72). The synergistic effects of these factors contribute to an accelerated postoperative recovery process. As an integrated outcome measure, the reduction in length of hospital stay reflects not only improvements in the quality of medical care but also demonstrates the comprehensive cascade of NST intervention effects (28, 29), spanning from initial nutritional optimization to ultimate clinical outcome improvements.

### 4.1 Limitation

Despite the positive results observed in this study, several limitations should be acknowledged. First, as a single-center study, the sample size was relatively limited, the study population consisted primarily of Asian patients from a single Chinese medical center, which may limit the generalizability of our findings to non-Asian populations, and acknowledge possible selection bias might exist and the generalizability of these findings requires validation in larger, multicenter studies. Second, owing to a pragmatic approach, the follow-up period in this study was relatively short, and the long-term effects of NST intervention on patient survival and quality of life remain to be further investigated. This study was conducted at a tertiary hospital with well-established NST infrastructure, and the feasibility and effectiveness of implementation in centers without specialized nutritional support teams require further validation. In the future, multicenter studies in different ethnic populations and healthcare resource settings are needed to further validate the broader applicability of our findings and to explore NST implementation models suitable for various healthcare institutions. Finally, the absence of a cost-effectiveness analysis precludes a comprehensive evaluation of the health economic value of the NST model.

## 5 Conclusion

This study demonstrates that systematic perioperative management by an NST in older adults malnourished patients with gastric cancer was associated with higher attainment of energy and protein targets, improvements in nutritional and immune function, lower postoperative 30-day mortality, and faster clinical recovery. In light of current guidelines and emerging evidence, NST-led nutritional intervention should be considered an essential component of perioperative care for older adults with malnutrition.

## Data Availability

The raw data supporting the conclusions of this article will be made available by the authors, without undue reservation.
