# Supplementary material for: Effectiveness of nutrition support team-led care on perioperative outcomes in malnourished older adults with gastric cancer
Source: Front Nutr. 2025 Dec 10;12:1707892. doi: 10.3389/fnut.2025.1707892 (PMC12729130; doi:10.3389/fnut.2025.1707892)
Supplement: Supplementary file 1 [file Table_1.docx]

Supplementary Table 1 Postoperative nutritional indicators

| Postoperative nutritional indicators | Time | TN  M (P_25_, P_75_). | NST  M (P_25_, P_75_). | *P* |
| --- | --- | --- | --- | --- |
| PNI | D3 | 44.60 (42.50, 47.55) | 45.9 (41.75, 49.20) | 0.05 |
|  | D7 | 43.30 (40.50, 46.40) | 46.6 (43.45, 50.05) | < 0.0001 |
|  | Before discharge | 43.10 (39.45, 45.95) | 47.30 (43.45, 50.05) | < 0.0001 |
| Prealbumin | D3 | 122.1 (96.10, 149.40) | 124.4 (100.90, 163.60) | 0.16 |
|  | D7 | 118.7 (91.40, 144.50) | 136.8 (123.10, 172.00) | < 0.0001 |
|  | Before discharge | 121.40 (92.30, 151.80) | 157.00 (128.20, 183.40) | < 0.0001 |
| Albumin | D3 | 34.50 (31.70, 37.40) | 35.30 (31.85, 38.10) | 0.09 |
|  | D7 | 34.00 (31.30, 36.90) | 34.6 (32.30, 37.35) | 0.06 |
|  | Before discharge | 34.10 (31.70, 37.40) | 35.20 (32.10, 38.45) | 0.06 |
| Hemoglobin | D3 | 105.00 (89.00, 122.00) | 104.00 (89.00, 122.00) | 0.75 |
|  | D7 | 105.00 (89.00, 122.00) | 104.00 (89.00, 121.50) | 0.94 |
|  | Before discharge | 103.00 (88.00, 117.00) | 103.00 (86.00, 120.5) | 0.56 |

Compared to the TN group, ^****^*P* < 0.0001
